# Occurrence of spontaneous bilateral tubal pregnancy in a low-income setting in rural Cameroon: a case report

**DOI:** 10.1186/s13104-017-3021-y

**Published:** 2017-12-04

**Authors:** Conrad S. Tankou, Carlson-Babila Sama, Jinette Lorraine Guedem Nekame

**Affiliations:** 1 Bambalang Medicalized Health Centre, Bambalang, Cameroon; 2 Galactic Corps Research Group (GCRG), Buea, Cameroon; 3 Department of Anesthesia and Reanimation, Catholic University of Central Africa, Yaounde, Cameroon

**Keywords:** Ectopic pregnancy, Bilateral tubal pregnancy, Cameroon

## Abstract

**Background:**

Ectopic pregnancy is a life-threatening emergency warranting immediate recognition and prompt intervention. Bilateral tubal pregnancy is the rarest form of ectopic pregnancy with very limited data on its occurrence reported in sub-Saharan Africa.

**Case presentation:**

We report the case of a 40-year-old multigravida with chief complains of lower abdominal pain evolving for 5 days in whom an intraoperative diagnosis of spontaneous bilateral tubal pregnancy (plus ruptured right tube) was made.

**Conclusions:**

Ectopic pregnancy is one of the major causes of maternal mortality in sub-Saharan Africa. Thus, clinicians should maintain a high index of suspicion, even in the absence of hallmark features and thorough clinical examination undertaken especially in resource-limited settings. Examination of both tubes at laparotomy for ectopic pregnancy should be routine and mandatory.

## Background

Globally, ectopic pregnancy remains the most common life-threatening emergency in early pregnancy, accounting for significant morbidity and mortality, especially in low-income settings. In the absence of iatrogenic ovulation induction or the use of assisted reproductive technologies, simultaneous bilateral tubal pregnancy (SBTP) represents the rarest form of extrauterine pregnancy with an estimated occurrence of one per every 200,000 live births [1]. To the best of our knowledge, we report the first case of SBTP occurring in a rural setting in Cameroon, with highlights on some diagnostic challenges.

## Case presentation

A 40-year-old lady (gravida 10 para 9) consulted at our emergency unit for a 5-day history of worsening lower abdominal pains. Prior to consultation, she had received some undocumented over-the-counter pain killers without significant relief of the pains. During her review, she denied any possibilities of being pregnant and reported to have experienced a recent 4-day vaginal bleed which she considered her normal menstrual flow as it was of similar characteristics to her periods. Her past history equally noted several episodes of sexually transmitted infections (STIs) which were treated, but no previous history of abdomino-pelvic surgery or abortion.

On entry she was haemodynamically stable but agitated. Examination of the abdomen revealed marked tenderness at the hypogastric region as well as both flanks. There were no signs of peritonism. On vaginal examination the cervix was closed, tender on transverse mobilisation (positive chandelier’s sign), and gloved fingers were not stained with blood. Our diagnosis at this point was a ruptured ectopic pregnancy which was supported by an abdominal paracentesis revealing a haemoperitoneum. About 6 mL of non-coagulating blood was aspirated. Laboratory exams revealed a positive pregnancy test (using the rapid urinary pregnancy test strips) and a haemoglobin level of 7.2 g/dL. Ultrasonography was not done due to limited resources.

**Fig. 1:**
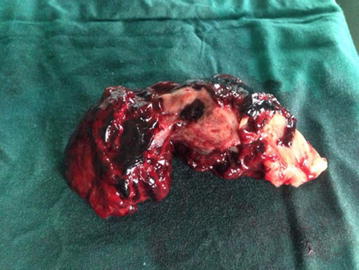
Ruptured right ectopic (tubal) pregnancy

**Fig. 2:**
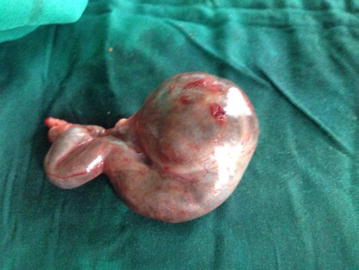
Unruptured left ectopic (tubal) pregnancy

Following an informed consent after counselling the patient and her husband, an emergency laparotomy was done. The intraoperative findings revealed a ruptured right tubal pregnancy and an intact left tubal pregnancy, with a haemoperitoneum of approximately 1500 mL. We performed a bilateral salpingectomy (Figs. 1 and 2). The patient was transfused two pints of group O rhesus positive blood in the immediate postoperative period. Post-operative course was uneventful, and the patient was discharged 6 days post-surgery.

## Discussion

Ectopic pregnancy is a complication in early pregnancy in which the embryo implants outside the normal uterine cavity. Ectopic pregnancies account for about 1–2% of all pregnancies and would often present as a surgical emergency in the majority of cases [2]. Several risk factors have been identified including a history of sexually transmitted infections; intrauterine device; smoking; hormonal contraception; pelvic surgery; ovulation induction and assisted reproductive technologies [3]. Over 90% of ectopic pregnancies implant in the fallopian tubes (tubal pregnancy). Other implantation sites include the cervix, ovaries and abdomen [4, 5].

Simultaneous bilateral tubal pregnancy (SBTP) is an extremely rare occurrence with its incidence somewhere between 1 per 125 and 1 per 1580 extrauterine pregnancies and in most cases, results from assisted reproduction techniques. More common are twin pregnancies in the same tube and heterotopic pregnancies. Theorised possible mechanisms for the occurrence of a bilateral tubal pregnancy are: simultaneous multiple ovulation; sequential impregnation; or transperitoneal migration of trophoblastic cells from one extrauterine pregnancy to the other tube with implantation there.

Our patient did not present with the classical features of ectopic pregnancy such as overt amenorrhea, spot vaginal bleeding and abdominal pain which may radiate to the shoulder (if there is peritoneal bleed secondary to rupture). Furthermore, considering the amount of intraabdominal haemorrhage encountered at laparotomy (~ 1500 cc); being haemodynamically stable at presentation only further illustrates some of the diagnostic challenges which may delay timely diagnosis of this surgical emergency with a resultant significant morbidity/mortality. These emphasises the need for a high index of suspicion and the invaluable role of thorough clinical examination especially in resource-constraint settings like ours where access to basic investigations including ultrasonography remains limited. Worthy of note is the fact that the diagnosis of bilateral tubal pregnancy is usually made intraoperatively, thus underscoring the paramount importance of identifying and closely examining both tubes at the time of surgery.

Owing to its rarity, there is no comprehensive clinical guideline or protocol for the management of SBTP. Management generally varies depending on the condition of the patient, extent of tubal damage and the desire for future fertility [6]. Various surgical treatment options have ranged from the extremes of a total abdominal hysterectomy (TAH) with bilateral salpingooophorectomy to the conservative approach, with laparoscopic techniques involving salpingectomy or salpingostomy as warranted. In low-income settings, open surgery (laparotomy) is still widely practiced over laparoscopic techniques.

## Conclusions

We have reported a case of spontaneous bilateral tubal pregnancy which has a very rare occurrence especially in the absence of ideal risk factors such as ovulation stimulation and assisted reproductive technology as noted in this case. Our report builds on literature especially in low-income settings where data on its occurrence is very limited. Giving that our patient did not present with classical features of ectopic pregnancy, it reiterates the invaluable need for clinicians to lay more emphasis on thorough clinical examination as access to basic para-clinical investigations in such settings may be limited. Examination of both tubes at laparotomy for ectopic pregnancy should be routine and mandatory.

## Abbreviations

STIs: sexually transmitted infections
SBTP: simultaneous bilateral tubal pregnancy
TAH: total abdominal hysterectomy
